# Lnc-STYK1-2 regulates bladder cancer cell proliferation, migration, and invasion by targeting miR-146b-5p expression and AKT/STAT3/NF-kB signaling

**DOI:** 10.1186/s12935-021-02114-4

**Published:** 2021-07-31

**Authors:** Ranran Dai, Qingping Jiang, You Zhou, Ruifeng Lin, Hai Lin, Yumin Zhang, Jinhu Zhang, Xingcheng Gao

**Affiliations:** 1 Guangdong Key Laboratory of Urology, Guangzhou Medical University, Guangzhou, China; 2 Department of Pathology, The Third Affiliated Hospital of Guangzhou Medical University, Guangzhou, China; 3 Department of Children’s Stomatology, Stomatology Hospital of Guangzhou Medical University, Guangzhou, China; 4 Department of Urology, Minimally Invasive Surgery Center, The First Affiliated Hospital of Guangzhou Medical University, 151 Yanjiang road, Yuexiu district, Guangzhou, 510120 China

**Keywords:** Lnc-STYK1-2, Bladder cancer, miR-146b-5p, Proliferation, Migration and invasion, ITGA2, AKT/STAT3/NF-kB

## Abstract

**Background:**

Epigenetic modulation by noncoding RNAs substantially contributes to human cancer development, but noncoding RNAs involvement in bladder cancer remains poorly understood. This study investigated the role of long noncoding RNA (lncRNA) lnc-STYK1-2 in tumorigenesis in cancerous bladder cells.

**Methods:**

Differential lncRNA and mRNA profiles were characterized by high-throughput RNA sequencing combined with validation via quantitative PCR. Bladder cancer cell proliferation was assessed through MTS, and bladder cancer cell migration and invasion were assessed through a Transwell system. The in vivo tumorigenesis of bladder cancer cells was evaluated using the cancer cell line-based xenograft model. The dual-luciferase reporter assay verified the association of miR-146b-5p with lnc-STYK1-2 and the target gene. Protein abundances and phosphorylation were detected by Western blotting.

**Results:**

Alterations in lncRNA profiles, including decreased lnc-STYK1-2 expression, were detected in bladder cancer tissues compared with adjacent noncancerous tissues. lnc-STYK1-2 silencing effectively promoted proliferation, migration, and invasion in two bladder cancer cell lines, 5637 and T24, and their tumorigenesis in nude mice. lnc-STYK1-2 siRNA promoted miR-146b-5p and reduced ITGA2 expression in bladder cancer cells. Moreover, miR-146b-5p suppressed ITGA2 expression in bladder cancer cells through direct association. Also, lnc-STYK1-2 directly associated with miR-146b-5p. Finally, miR-146b-5p inhibitors abrogated the alterations in bladder cell functions, ITGA2 expression, and phosphorylation of AKT, STAT3, and P65 proteins in 5637 and T24 cells induced by lnc-STYK1-2 silencing.

**Conclusion:**

lnc-STYK1-2 inhibited bladder cancer cell proliferation, migration, and tumorigenesis by targeting miR-146b-5p to regulate ITGA2 expression and AKT/STAT3/NF-kB signaling.

## Background

Bladder cancer is a common human malignant disorder that originated from the epithelium covering the bladder inner surface and is well known for its high prevalence, mortality, morbidity, and poor prognosis [1, 2]. Globally, more than 420,000 new cases of bladder cancer have been estimated each year, with more than 160,000 cancer deaths due to bladder cancer, especially in the male population and those living in underdeveloped countries [3]. So far, the clinical management of bladder cancer patients mainly depends on transurethral resection, intravesical chemotherapy, radical cystectomy, or neoadjuvant chemotherapy, which usually produces limited therapeutic effects and low survival rates in those with distant metastasis [4]. The initiation and progression of bladder cancer are mediated by alterations in multiple signaling cascades, such as Akt (protein kinase B), STAT3 (signal transducer and activator of transcription), and NF-κB (nuclear factor-kappa B) [5, 6]. However, little is known about the molecular mechanisms regulating their activation during bladder cancer development.

Long noncoding RNAs (lncRNAs) are a huge group of transcripts in a size of more than 200 nt without protein-coding functions. They are prevalently expressed in various tissues and cell types in almost all species [7–9]. It has been extensively documented that lncRNAs could substantially regulate gene expression by modifying chromosome status, interference with spliceosome activity, competing with other noncoding RNAs as a competing endogenous RNA (ceRNA), or modulation of mRNA stability and translation [7]. Hence, the differential expression of lncRNAs plays an essential role in the pathogenesis of many human diseases, such as diabetic nephropathy, vascular diseases, and multiple cancers [8–11]. In bladder cancer cells, the expression profiles of lncRNAs were significantly altered with a high correlation with cancer formation, development, metastasis, and 5-year survival rates [12, 13]. For instance, the epithelial–mesenchymal transition process in bladder cancer cells was recently reported to be greatly promoted by the lncRNA H19 by changing DNA methyltransferase 3B (DNMT3B) expression [14]. Moreover, proliferation rates and metastasis of bladder cancer cells could be substantially regulated by the lncRNA SPRY4-IT1 (SPRY4 intronic transcript 1), mediated by its impact on the expression of enhancer of zeste homolog 2 (EZH2) [15]. The pathogenic roles of new lncRNAs in bladder cancer development need further elucidation, considering the many differentially expressed lncRNAs in bladder cancer cells.

As introduced above, one major mechanism of lncRNAs in regulating the functional gene expression is acting as ceRNAs to regulate the expression and activity of other noncoding RNAs, such as microRNAs (miRNAs) [16, 17]. For instance, developing large B cell lymphoma and its sensitivity to immunotherapy was regulated by the lncRNA SNHG14 (small nucleolar RNA host gene 14) via its targeting of miR-5590-3p and the resultant expressional alteration in ZEB1 (zinc finger E-box binding homeobox 1) [18]. Hepatic cancer cell growth could also be modulated by the interaction between the lncRNA ZFAS1 (zinc finger antisense 1) and the miR-193a-3p, leading to changes in the hepatocyte growth factor (HGF) signaling pathway [19]. Additionally, the miR-146b-5p could also be sponged by the lncRNA SOX2-OT (SOX2 overlapping transcript) in nasopharyngeal carcinoma cells, resulting in the altered proliferation and metastasis of cancer cells [20]. In bladder cancer tissues, the expression of miR-146b-5p was significantly increased compared with that in noncancerous bladder tissue, contributing to the invasive properties of bladder cancer cells by regulating ETS2 (erythroblastosis virus oncogene homolog 2) and MMP2 (matrix metalloproteinase 2) expression [21]. However, whether lncRNAs could also target miR-146b-5p has never been previously explored.

This study investigated the epigenetic events associated with bladder cancer development by characterization of differentially expressed lncRNAs between bladder cancer tissues and adjacent noncancerous tissues in patients. Also, the pathogenic roles of lnc-STYK1-2 in bladder cancer were further studied using in vitro and in vivo assays, followed by identifying its interacting microRNAs and downstream signaling pathways. These results would provide new insights into the molecular mechanism driving the initiation and progression of bladder cancer, which might serve as new targets for early detection and clinical management of bladder cancer patients.

## Material and methods

### Clinical tissue collection

The bladder cancer tissues were surgically collected from patients diagnosed with bladder cancer after a pathological examination. Inclusion criteria are as follows: (1) no tumor history was found and (2) patients had no chemotherapy, radiotherapy, or any anti-tumor treatment. Patients underwent surgical treatment at the Third Affiliated Hospital of Guangzhou Medical University (Guangzhou, China) between October 2016 and October 2019. Each patient provided written consent before the surgical operation. The Medical Ethics Committee of the Third Affiliated Hospital of Guangzhou Medical University approved the whole research plan in advance. Adjacent noncancerous bladder tissue samples collected from the same patients were used as the control. All freshly collected tissues were immediately stored in liquid nitrogen for subsequent assays.

### Transcriptome difference analysis

The differentially expressed lncRNAs and mRNAs in bladder cancer tissues collected from patients or cultured cancerous cell lines were identified using the deep sequencing method. The preparation of total RNA samples from bladder tissue or cell lines was briefly performed using Trizol Reagent (#15596-026; Invitrogen, USA) according to the manufacturer’s instructions. Subsequently, removing ribosomal RNA (rRNA) from total RNA samples was done using the Ribo-zero rRNA removal reagents (Epicentre, USA) as instructed by the producer. Then, the RNA sequencing library was constructed using the NEBNext Ultra II Directional RNA Library Prep Kit (for Illumina; #E7760S; New England Biolabs) following the producer’s instructions. Library was subjected to sequencing on the Illumina Hiseq 4000 system (Illumina, USA). Raw reads produced using the sequencing procedure were further filtered by removing low-quality reads and reads containing poly-N or adaptors. Clean reads in the FASTQ format were subsequently subjected to the calculation of the RPKM (reads per kilobase per million mapped reads) values according to statistical routines provided by the Cuffdiff software. The combination of log2 ratio (≥ 1) and fold change ratio (FDR) value (≤ 0.001) was applied for defining the differential expression of lncRNAs and mRNAs between groups.

### Bioinformatic analysis

The scatterplot representing the differential expression of lncRNAs between bladder cancer tissues and adjacent noncancerous bladder tissue, and the hierarchical clustering analysis of lncRNAs or mRNAs were established based on the RPKM values using the R language software. Enrichment of differentially expressed mRNAs in KEGG (Kyoto Encyclopedia of Genes and Genomes) signaling pathways were analyzed using DAVID (Database for Annotation, Visualization, and Integrated Discovery). The potential miRNAs targeted by lnc-STYK1-2 and the lncRNA–miRNA–mRNA interaction networks based on differentially expressed mRNAs were constructed using the Cytoscape software (v.3.8.2) as previously introduced [22].

### Quantitative RT-PCR

Total RNA samples from bladder tumor tissues or cell lines were extracted as introduced above, whose concentrations were determined on a NanoDrop 2000 device (Thermo Fisher Scientific). Approximately 1.5-μg RNA samples in each group were used to synthesize cDNAs catalyzed using the M-MLV RT kit (#M1701; Promega) following the manufacturer’s instructions. Relative expression levels of lnc-STYK1-2 and miR-146b-5p were finally measured using the quantitative PCR method for lnc-STYK1-2 using the Platinum II Hot-Start Green PCR Master Kit (#14001013, Thermo Fisher) and for miR-146b-5p using the miScript II RT Kit (#218161, Qiagen), according to the manufacturer’s instructions. Quantitation was performed using three biological replicates, and GAPDH and U6 expression levels were detected as the internal standard for lnc-STYK1-2 and miR-146b-5p, respectively. The sequences of primers used for expressional determination are listed in Table 1.

**Table 1:** The sequences of primers applied for quantitative RT-PCR

Primers name	Sequences (5′-3′)
lnc-STYK1-2-F	CCTTGGCACTGTCAAATGGTTC
lnc-STYK1-2-R	ACACACCCTCTCCTCACATAGC
Hsa-miR-146b-5p-RT	GTCGTATCCAGTGCAGGGTCCGAGGTATTCGCACTGGATACGACACAGCC
Hsa-miR-146b-5p-F	CGGAGCACTTGAGAACTGAAT
Fas-F	TGTGTGATGAAGGACATGGC
Fas-R	ACATTTGGTGCAAGGGTCAC
NR5A2-F	AGAAGCCATGTCTCAGGTGATC
NR5A2-R	AAGGCAGCATGGTTCAGAGG
ITGA2-F	CCGATGTGTCTATTGGTGCCT
ITGA2-R	GGTCTGAACTTTGCACTGAAGC
GAPDH F	GAGTCAACGGATTTGGTCGT
GAPDH R	GACAAGCTTCCCGTTCTCAG
U6-F	CTCGCTTCGGCAGCACA
U6-R	AACGCTTCACGAATTTGCGT

### Cell culture and transfection

The bladder cancer cell lines UM-UC-3 (#CC1003), TCCSUP (#CC1006), 5637 (#CC1002), and T24 (#CC1001) and human embryonic kidney cells HEK-293T (#CC4003) were purchased from the CellCook (Guangzhou, China), which were authenticated using the short tandem repeat profiling method. Cells were cultured in Dulbecco's modified Eagle’s medium (DMEM; Thermo Fisher Scientific) supplemented with 10% FBS (Thermo Fisher Scientific) and penicillin/streptomycin at 37 °C in a humidified culture chamber with 5% CO_2_. The si-lnc-STYK1-2 (sense: 5′-GGGUGUGUAAUCUUCUGUC-3′, antisense: 5′-GACAGAAGAUUACACACCC-3′) and negative control sequences (sense: 5′-GACAGAAGAUUACACACCC-3′, antisense: 5′-GGGUGUGUAAUCUUCUGUC-3′), miR-146b-5p inhibitor, and mimics, were synthesized by GenePharma (Shanghai, China). For dual-luciferase reporter assay, wild-type lnc-STYK1-2 and mutant-type lnc-STYK1-2 were synthesized by General Biol (Anhui, China). Then, Lipofectamine 3000 Reagent (Thermo Fisher Scientific) was used for transfection according to the manufacturer’s instructions.

### Construction of stable strain sh-lnc-STYK1-2

Lentivirus of lnc-STYK1-2 was provided by the GenePharma (Shanghai, China). Then, the stable strain sh-lnc-STYK1-2 was constructed following the instruction of lentivirus.

### Cell proliferation

The proliferation rates of bladder cancer cells were detected using the colorimetric MTS assay kit (#ab197010) following the manufacturer’s instructions. Briefly, bladder cancer cells were seeded in 96-well microtiter plates (5 × 10^3^ cells/well) after treatment and incubated with 20-µL MTS Reagent for 2 h at 37 °C. The proliferation of cultured bladder cancer cells was then evaluated by measuring the OD490 values using a microplate reader (Thermo Fisher Scientific, USA) following gentle shaking at a specific time. Finally, three biological replicates were done for comparing cell proliferation rates.

### Cell migration and invasion

The migration and invasion of bladder cancer cells were analyzed using the Transwell system (Corning, NY, USA). Briefly, bladder cancer cells (1 × 10^5^ cells/ml) were seeded in the upper chamber of Tranwell plates containing serum-free DMEM, whereas the lower chambers were filled with DMEM with 10% FBS. Cells that migrated to the lower chambers after normal culture for 48 h were stained with 1% crystal violet solution for 12 min and photographed under microscopy to assess the migration. To evaluate the invasive capacity, bladder cancer cells were seeded in Transwell plates pre-coated with Matrigel matrix (Corning, NY, USA) following the same experimental procedures.

### In vivo tumorigenesis

Six-week-old female BALB/c nude mice were purchased from the Guangdong Medical Experimental Animal Center and maintained in a pathogen-free experimental facility with free access to food and drinking water. The Forevergen Biosciences Experimental Animal Ethics Committee approved all experiments in advance. First, the tumorigenesis of bladder cancer cells was assessed through the cancer cell line-based xenograft (CDX) as previously described [23]. Briefly, the cultured bladder cancer cells after specified transfection were introduced into the nude mouse rear flank by subcutaneous injection (2 × 10^6^ cells/mouse). Finally, the size and weights of tumors formed in nude mice were measured 30 days after cell injection.

### Dual-luciferase reporter assay

The dual-luciferase reporter assay kit (#E1910) produced by the Promega company was used to validate the binding of miR-146b-5p with lnc-STYK1-2 in HEK-293T cells. Briefly, the lnc-STYK1-2 wild-type (WT) sequences (5′-GACGCGGAGAAAAAAGTTCTCG-3′) or its mutant (MUT) version (5′-GACGCGGAATTAAATAGGAGAG-3′) ligated with the pmirGLO vectors were introduced into the cultured HEK-293T cells using the Lipofectamine 3000 Reagent (Thermo Fisher Scientific) following the manufacturer’s instructions. Meanwhile, the hsa-miR-146b-5p mimics or their negative control were transfected into the abovementioned HEK-293T cells. Finally, HEK-293T cells were lysed, and the luciferase activities in cell lysates were measured using a GloMax-20/20 luminometer (Promega, Madison, USA) to evaluate the lncRNA and miRNA interaction.

### Western blotting

Total proteins in cultured bladder cancer cells were prepared by lysis using the Cell Total Protein Lysis kit (Sangon Biotech, Shanghai, China), following the producer’s instructions. The protein concentration was detected using the BCA method. Then, the protein was boiled at 100 °C for 5 min, separated by sodium dodecyl sulfate–polyacrylamide gel electrophoresis, and transferred onto a polyvinylidene fluoride (PVDF) membrane pre-immersed with methanol. Following blocking in 5% bovine serum albumin solution for 2 h at room temperature, PVDF membranes were incubated with primary antibodies at 4 °C overnight and with secondary antibodies for 1–2 h at room temperature. The relative protein levels were finally detected by development with enhanced ECL substrate solutions (#32106; Thermo Fisher Scientific). The bands were observed using Tanon-5200CE (Biotanon, Shanghai, China). The expression of GAPDH protein was simultaneously analyzed as the internal standard for non-phosphorylation proteins. Primary antibodies used in Western blotting include anti-ITGA2 (#BM5058; Boster; 1:1000), anti-p-AKT (#4060S; CST; 1:1000), anti-AKT (#2920S; CST; 1:1000), anti-p-p65 (#3033S; CST; 1:1000), anti-p65 (#ab7970; Abcam; 1:1000), anti-p-STAT3 (#9145; CST: 1:1000), anti-STAT3 (#ab31370; Abcam; 1:1000), and anti-GAPDH (#60004-1-Ig; Proteintech; 1:1000).

### Statistical analysis

The quantitative results in this study presented as mean ± standard deviation were evaluated for statistical significance using the SPSS v.20.0 (IBM, Armonk, NY, USA). The significances in differences between two or more groups were assessed using Student’s *t*-test or analysis of variance tests. *P* < 0.05 was used to define significant differences.

## Results

### Altered lncRNA profiles and decreased lnc-STYK1-2 expression in bladder cancer tissues

**Fig. 1:**
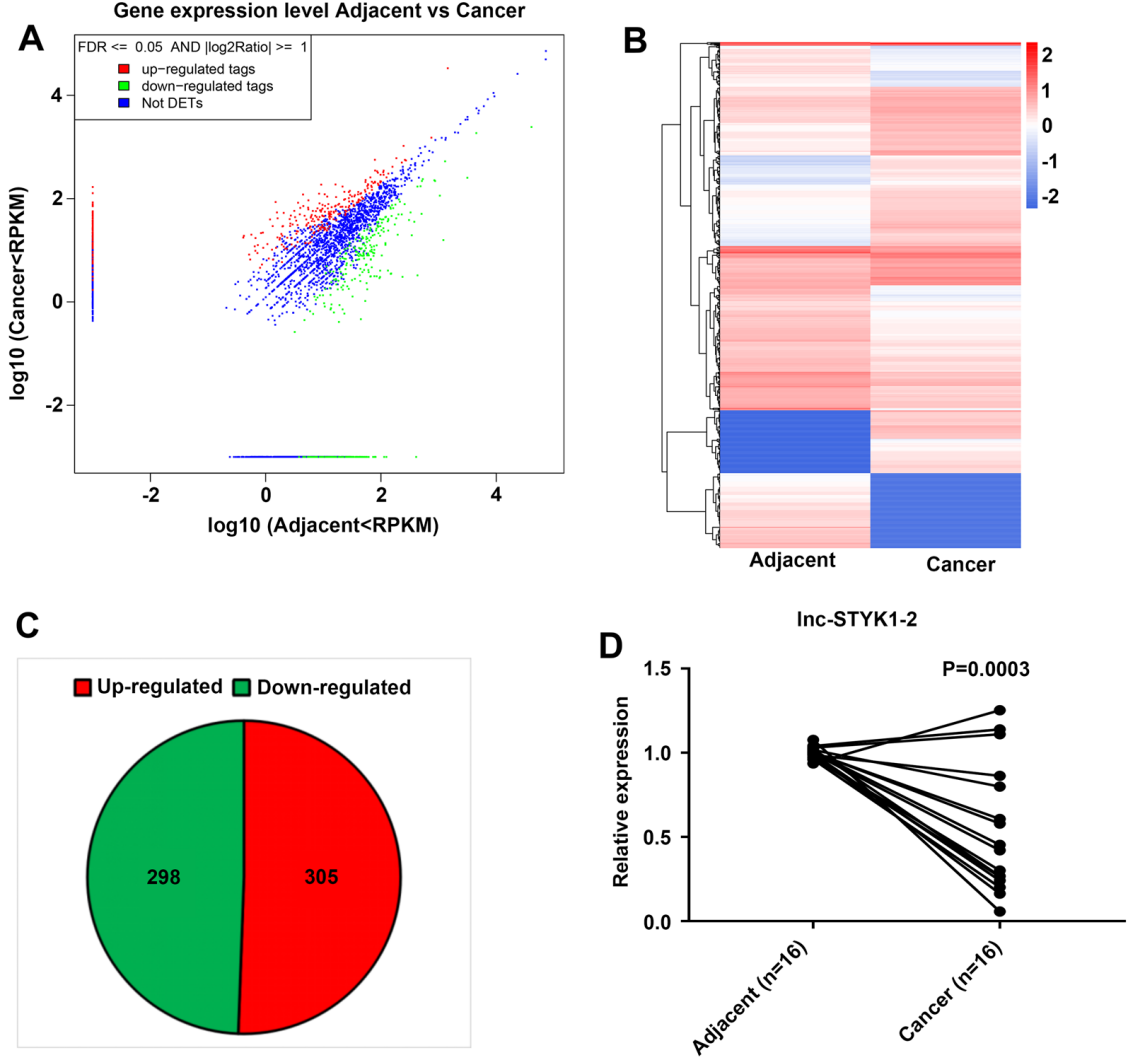
Significant lncRNA profile changes and downregulation of lnc-STYK1-2 expression in bladder cancer tissues. **A** A scatterplot showing the differential expression of lncRNAs between bladder cancer tissues and corresponding adjacent noncancerous bladder tissues. The expression of lncRNA between bladder cancer tissues and adjacent noncancerous bladder tissues was detected by high-throughput RNA sequencing. Upregulated, downregulated, and nonaltered lncRNAs are presented as red, green, and blue spots in the plot, respectively. **B** Hierarchical clustering of differentially expressed lncRNAs between bladder cancer tissues and the corresponding adjacent noncancerous bladder tissues. Relative higher and lower lncRNA expressions are indicated by red and blue colors, respectively. **C** Venn plots showing the numbers of significantly upregulated and downregulated lncRNAs between bladder cancer tissues and corresponding adjacent noncancerous bladder tissues. **D** Relative expression of lnc-STYK1-2 in cancerous and adjacent noncancerous bladder tissue samples collected from 16 patients. The lnc-STYK1-2 expression levels in bladder tissues were detected by quantitative RT-PCR. RPKM: reads per kilobase per million mapped reads; FDR: fold change ratio; DETs: differentially expressed transcripts; STYK1: serine/threonine/tyrosine kinase 1

To investigate the potential roles of lncRNAs in bladder cancer pathogenesis, we first characterized the differentially expressed lncRNAs in bladder cancer tissues collected from three bladder cancer patients. The RNA from these three tissues was mixed before high-throughput RNA sequencing, using their corresponding adjacent noncancerous bladder tissue as the controls (Fig. 1A–C). The scatterplot based on the RPKM values of lncRNA expression levels disclosed significant alterations in lncRNA profiles between the bladder cancer and adjacent tissues in cancer patients (Fig. 1A). The expression of 603 lncRNAs was significantly changed in bladder cancer tissues compared with adjacent tissues (FDR < 0.001; log2 ratio > 1 or <  − 1), including 298 downregulated and 305 upregulated lncRNAs (Fig. 1B and 1C). Among them, we observed that the expression of lnc-STYK1-2 in bladder cancer tissues was greatly downregulated compared with that in adjacent tissues, which were further validated by quantitative RT-PCR (qRT-PCR) in another 16 bladder cancer tissues (Fig. 1D). These results showed great alterations in lncRNA profiles and a decrease in lnc-STYK1-2 expression in bladder cancer tissues.

### lnc-STYK1-2 silencing promotes bladder cancer cell proliferation, migration, and invasion

**Fig. 2:**
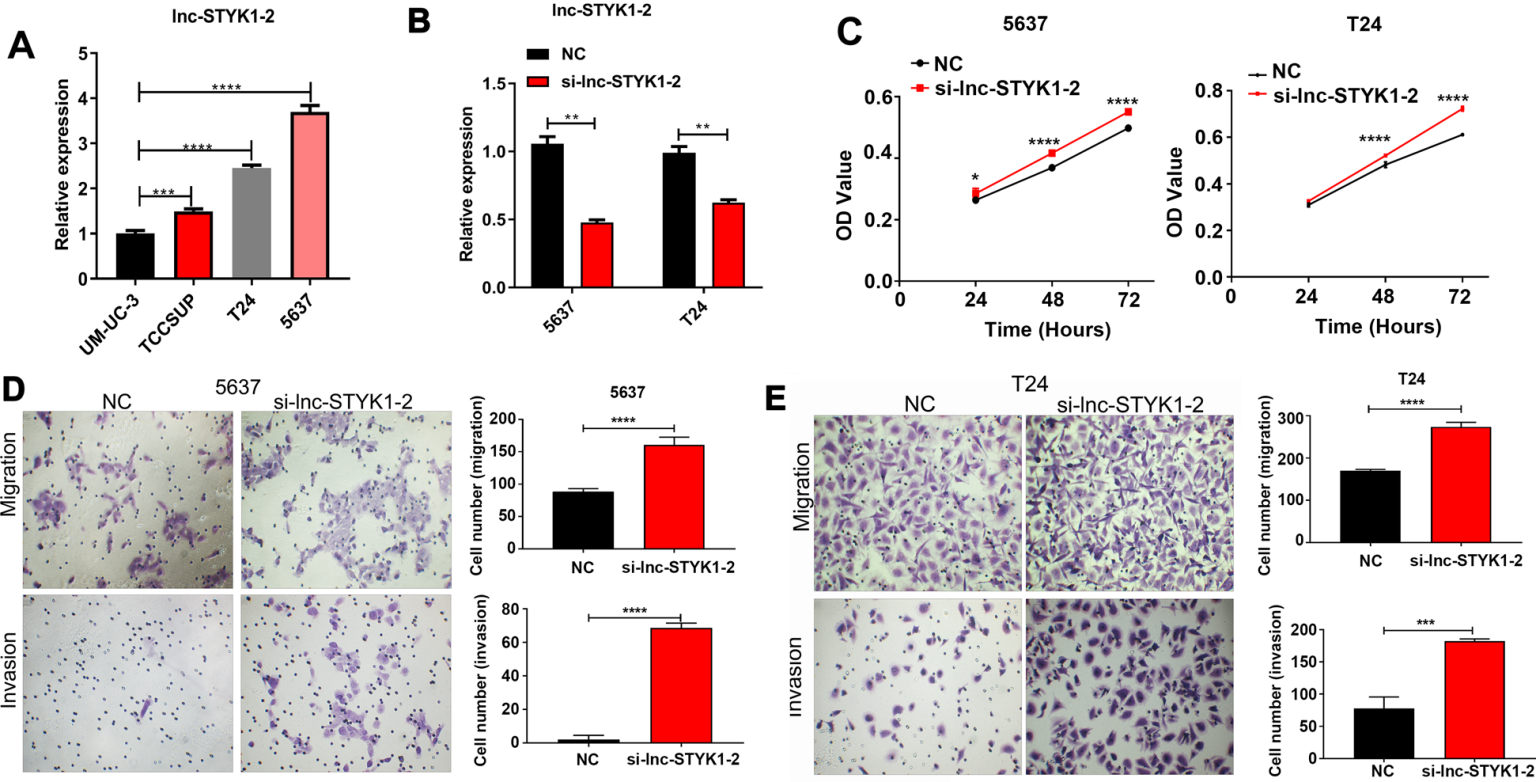
Promotion of bladder cancer cell proliferation, migration, and invasion by lnc-STYK1-2 silencing. **A** Relative expression levels of lnc-STYK1-2 in different bladder cancer cell lines. The expression of lnc-STYK1-2 in bladder cancer cell lines UM-UC-3, TCCSUP, T24, and 5637 was measured by quantitative RT-PCR. **B** Silenced lnc-STYK1-2 expression in T24 and 5637 cells by transfection with siRNAs targeting lnc-STYK1-2. The expression of lnc-STYK1-2 in T24 and 5637 cells was assessed by quantitative RT-PCR. **C** The alterations in T24 and 5637 cell proliferation induced by lnc-STYK1-2 silencing. Bladder cancer cell proliferation was evaluated through the MTS assay. **D**, **E** Enhanced migration and invasion capacities of bladder cancer cell lines by lnc-STYK1-2 silencing. The migration (**D**) and invasion (**E**) capacities of T24 and 5637 cells were detected using the Transwell system following lnc-STYK1-2 silencing. STYK1: serine/threonine/tyrosine kinase 1; NC: negative control; si-lnc-STYK1-2: siRNAs targeting lnc-STYK1-2; *P < 0.05; **P < 0.01

To further validate the function of differential expression of lnc-STYK1-2 in bladder cancer, we first detected its expression in multiple bladder cancer cell lines. We found that the bladder cancer cell lines T24 and 5637 possessed relatively higher levels of lnc-STYK1-2 expression (Fig. 2A). For the analysis of cellular functions of lnc-STYK1-2 during cancer development, we then knocked down the expression of lnc-STYK1-2 in T24 and 5637 cells by transfection with siRNA targeting lnc-STYK1-2; then, the results of qRT-PCR showed that the expression of lnc-STYK1-2 was successfully silenced (Fig. 2B). Furthermore, through the MTS method, we showed that the proliferation rates of T24 and 5637 cells were significantly promoted by siRNA-mediated lnc-STYK1-2 silencing, compared with the negative control groups (Fig. 2C). Subsequently, we analyzed the migration of T24 and 5637 cells with lnc-STYK1-2 silencing using the Transwell system. We found that their migration rates were also greatly enhanced by silencing of lnc-STYK1-2 expression compared with their negative controls (Fig. 2D). Similarly, the invasion capacities of T24 and 5637 cells were also remarkably promoted by lnc-STYK1-2 silencing compared with the negative control groups (Fig. 2E). These results showed that silencing of lnc-STYK1-2 expression could effectively promote the proliferation, migration, and invasion functions of bladder cancer cells.

### lnc-STYK1-2 silencing enhanced the tumorigenesis of bladder cancer cells

**Fig. 3:**
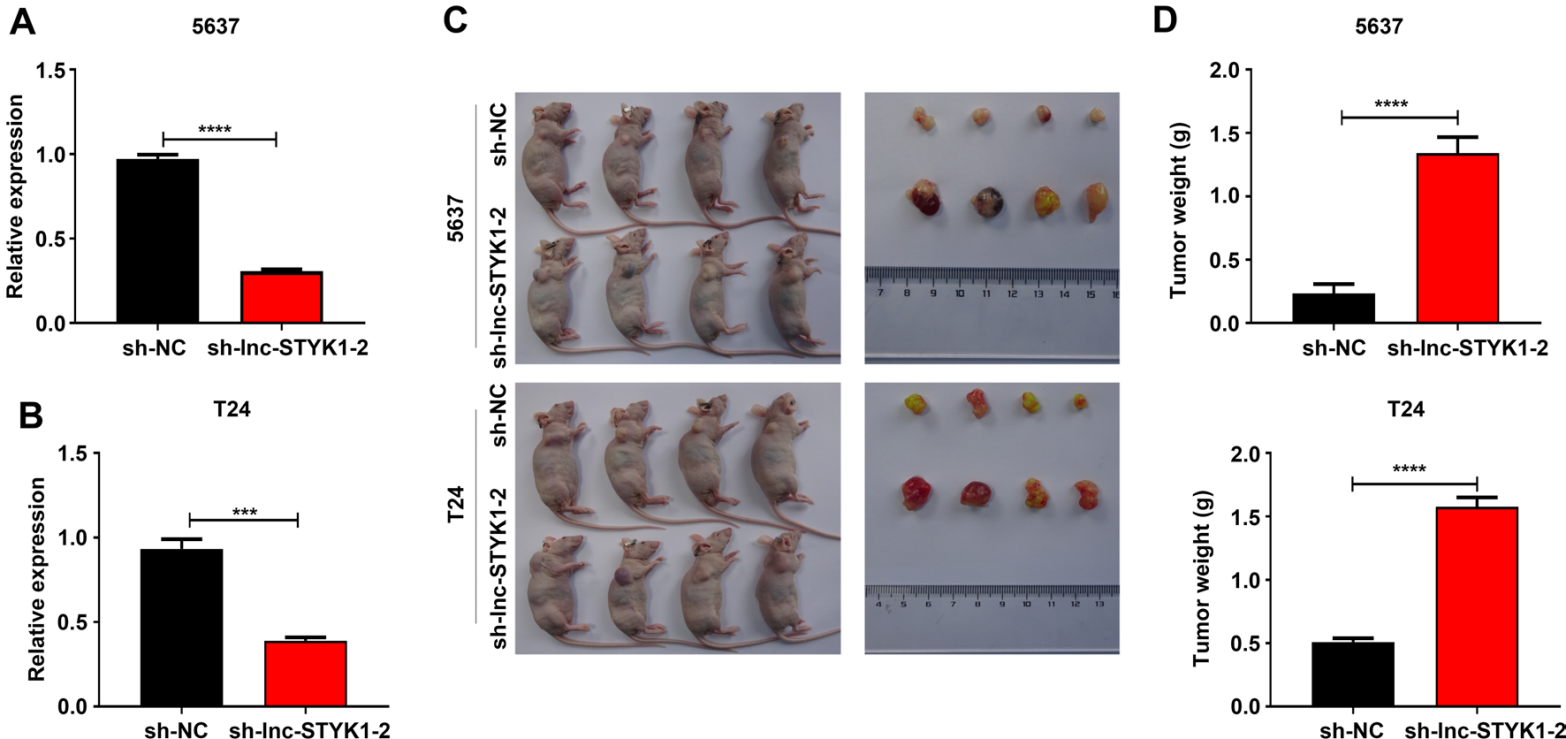
Enhanced tumorigenesis of bladder cancer cells by lnc-STYK1-2 silencing. **A**, **B** Significant suppression of lnc-STYK1-2 expression in the T24 and 5637 cells by lentivirus-mediated infection with shRNAs targeting lnc-STYK1-2. The expression of lnc-STYK1-2 in T24 and 5637 cells was detected by quantitative RT-PCR following recombinant lentivirus infection. **C** Greatly decreased sizes of tumors formed in nude mice injected with T24 and 5637 cells with lnc-STYK1-2 silencing. The tumors formed in mice were surgically collected 30 days after the injection of bladder cancer cells. **D** Quantitative analysis of the fresh weights of tumors originating from T24 and 5637 cells, with lnc-STYK1-2 silencing in nude mice. Sh: shRNA; NC: negative control; STYK1: serine/threonine/tyrosine kinase 1; ***P < 0.001

For further validation of the tumorigenic roles of lnc-STYK1-2, we then established the T24 and 5637 cells with stably silenced lnc-STYK1-2 expression using the lentivirus transfection system. The significant downregulation of lnc-STYK1-2 expression in T24 and 5637 cells caused by infection with recombinant lentivirus vectors ligated with shRNA sequences was first confirmed by qRT-PCR. The results showed that the expression of lnc-STYK1-2 was successfully interfered with by the lentivirus (Fig. 3A and B). Subsequently, the tumorigenesis capacity of T24 and 5637 cells was assessed using the CDX. The sh-lnc-STYK1-2 and sh-NC stable cells of T24 and 5637 were injected into nude mice to induce tumor formation (Fig. 3C). One month after the cancer cell injection, we showed that tumors derived from both T24 and 5637 cells with sh-lnc-STYK1-2 in nude mice were significantly larger than those in the sh-NC group (Fig. 3C). Consistently, the fresh weights of tumors formed in nude mice injected with T24 and 5637 cells were greatly increased by sh-lnc-STYK1-2, compared with those in the negative control groups (Fig. 3D). This in vivo assay persuasively showed that silencing of lnc-STYK1-2 expression could significantly enhance the tumorigenic capacities of bladder cancer cells.

### lnc-STYK1-2 regulates miR-146b-5p and ITGA2 expression in bladder cancer cells

**Fig. 4:**
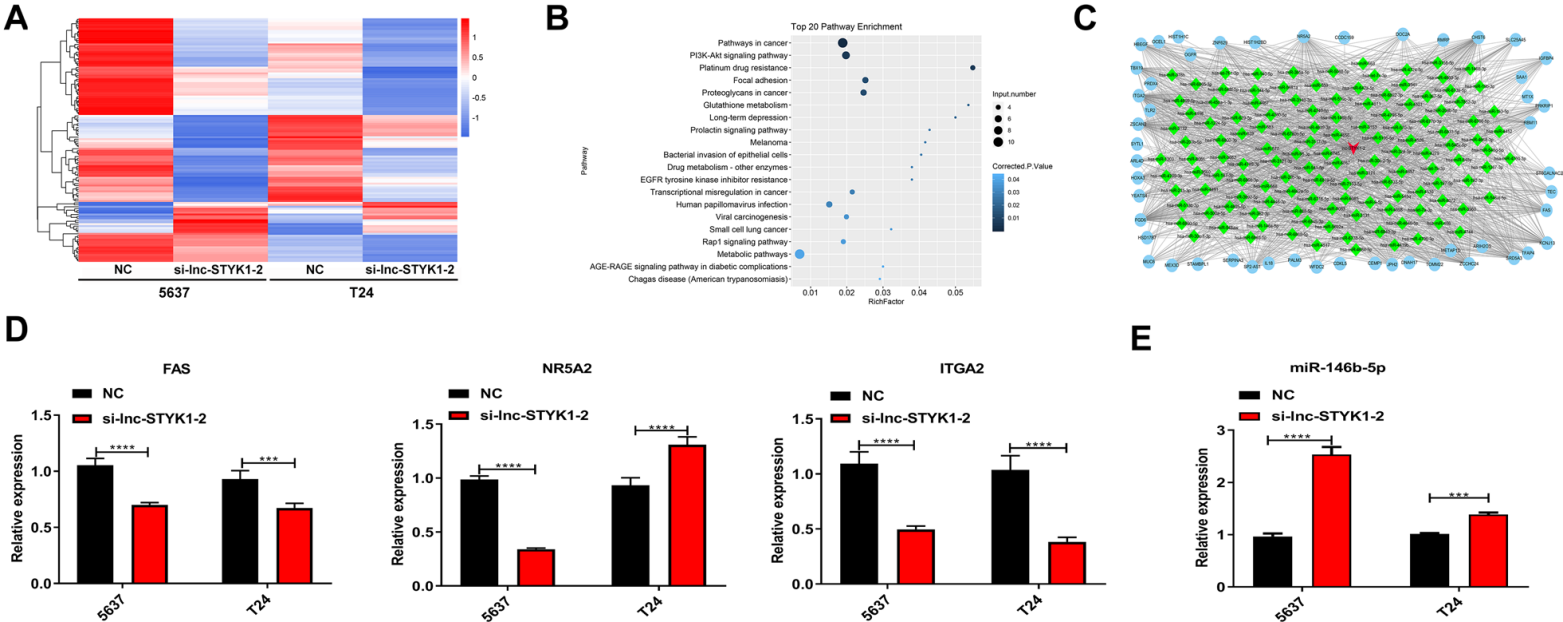
Regulation of miR-146b-5p and ITGA2 expression in bladder cancer cells by lnc-STYK1-2. **A** Hierarchical clustering of mRNAs differentially expressed in 5637 and T24 cells transfected with lnc-STYK1-2 siRNAs. The mRNA profile in bladder cancer cells was determined by RNA sequencing. Higher and lower mRNA expressions are presented in red and blue colors, respectively. **B** Functional categorization of differentially expressed mRNAs induced by lnc-STYK1-2 siRNAs based on KEGG signaling pathways. **C** The lncRNA–microRNA–mRNA interaction networks were established based on lnc-STYK1-2 and differentially expressed mRNAs in (A) through the Cytoscape software. **D** Expressional alterations in Fas, NR5A2, and ITGA2 genes in bladder cancer cells with silencing of lnc-STYK1-2. The relative gene expression was detected via qRT-PCR. **E** Increased expression of miR-146b-5p in T24 and 5637 cells caused by lnc-STYK1-2 silencing. NC: negative control; STYK1: serine/threonine/tyrosine kinase 1; NR5A2: nuclear receptor 5A; ITGA2: integrin α2; **P < 0.01; ***P < 0.001

To explore the molecular mechanisms underlying lnc-STYK1-2-regulated cancer development, we then analyzed the differentially expressed genes in 5637 and T24 cells with silenced lnc-STYK1-2 expression by RNA sequencing (Fig. 4A). One hundred and twenty-six genes were significantly regulated in the T24 and 5637 cells by lnc-STYK1-2 silencing (FDR < 0.001; log2 ratio > 1 or <  − 1), containing 109 downregulated and 17 upregulated mRNAs in lnc-STYK1-2-silenced bladder cancer cells compared with the negative control groups (Fig. 4A). Through KEGG categorization, these differentially expressed genes were significantly enriched in multiple cancer-related signaling pathways, such as the PI3K-AKT (protein kinase B) and Rap1 signaling pathways (Fig. 4B). To identify microRNAs involved in these processes, we subsequently established the lncRNA–miRNA–mRNA interaction networks by bioinformatic prediction based on lnc-STYK1-2 and differentially expressed mRNAs in 5637 and T24 cells with lnc-STYK1-2 silencing (Fig. 4C). In addition, we found that lnc-STYK1-2 might interact with miR-146-5p and other microRNAs to influence the expression of multiple signaling component proteins (Fig. 4C).

To validate the results obtained from the above transcriptome and bioinformatic analysis, we confirmed the expression of three candidate genes shown in Fig. 4C, namely, Fas, NR5A2 (nuclear receptor 5A), and ITGA2 (Integrin α2) (Fig. 4C). Through the qRT-PCR method, we found that the expression of Fas and ITGA2 genes were significantly downregulated in T24 and 5637 cells transfected with lnc-STYK1-2 siRNA, which agrees with the transcriptome analysis results. However, NR5A2 expression was significantly downregulated in 5637 cells and was significantly upregulated in T24 cells (Fig. 4D). Furthermore, ITGA2 exhibited the most significant decrease in T24 and 5637 cells induced by lnc-STYK1-2 silencing (Fig. 4D). Since ITGA2 was predicted to interact with miR-146b-5p in our lncRNA–miRNA–mRNA interaction networks (Fig. 4D), we then analyzed the expression of miR-146b-5p by qRT-PCR. We found that miR-146b-5p expression in the T24 and 5637 cells was greatly upregulated by lnc-STYK1-2 silencing (Fig. 4E). These results suggested an lnc-STYK1-2/miR-146b-5p/ITGA2 axis, which is involved in bladder cancer development.

### miR-146b-5p targets and suppresses ITGA2 expression in bladder cancer cells

**Fig. 5:**
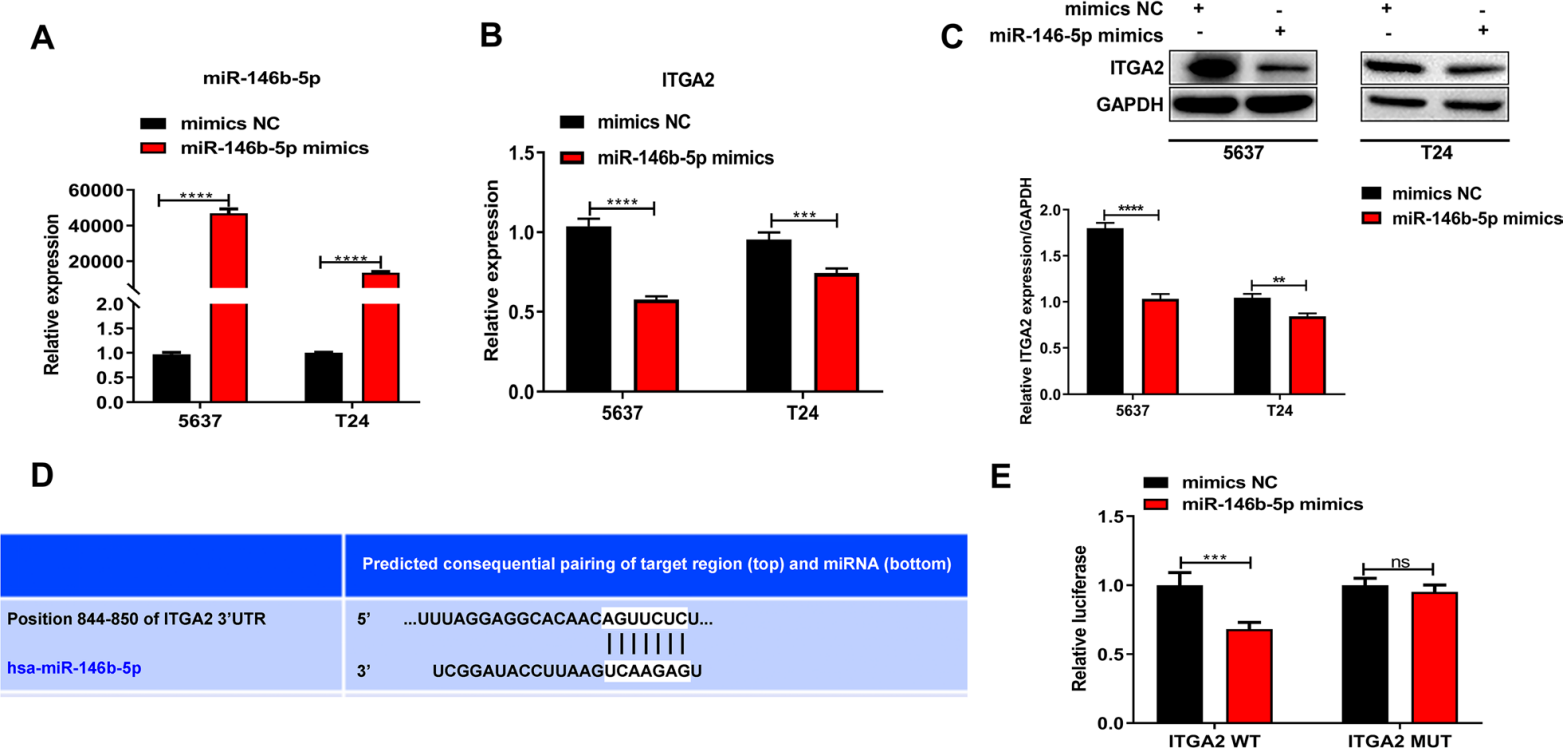
miR-146b-5p suppresses ITGA2 expression in bladder cancer cells by direct binding. **A**, **B** Relative expression of miR-146b-5p and ITGA2 in bladder cancer cells transfected with specific mimics targeting miR-146b-5p. The expression of miR-146b-5p (**A**) and ITGA2 (**B**) was detected via qRT-PCR. **C** Decrease in ITGA2 protein levels in bladder cancer cells induced by miR-146b-5p mimics. ITGA2 protein contents were analyzed by Western blotting. **D** The predicted binding sites between miR-146b-5p and the 3′ UTR region of the ITGA2 gene by bioinformatics. **E** The direct association of miR-146b-5p with ITGA2 gene 3′ UTR sequences in 293T cells. A dual-luciferase reporter assay was performed to validate the microRNA and gene sequence interaction. NC: negative control; ITGA2: integrin α2; GAPDH: glyceraldehyde-3-phosphate dehydrogenase; UTR: untranslated region; WT: wild type; MUT: mutant type; ***P < 0.001; ****P < 0.0001

To further clarify the relationship between miR-146b-5p and ITGA2 in bladder cancer cells, we then overexpressed miR-146b-5p in bladder cancer cells by transfection with miR-146b-5p mimics (Fig. 5A). We showed that miR-146b-5p mimics caused a significant increase in miR-146b-5p expression in the T24 and 5637 cell lines (Fig. 5A). Moreover, we observed that the expression of the ITGA2 gene in the T24 and 5637 cells was greatly reduced by miR-146b-5p overexpression (Fig. 5B). Also, downregulation of ITGA2 gene expression in T24 and 5637 cells due to miR-146b-5p mimics was further validated by Western blotting (Fig. 5C). The miR-146b-5p was predicted to bind with the 3′ UTR region of the ITGA2 gene sequences through bioinformatic analysis (Fig. 5D). Subsequently, we performed a dual-luciferase reporter assay to test the association of miR-146b-5p with ITGA2 gene sequences. We found that miR-146b-5p mimics induced a great decrease in the luciferase activity in 293T cells transfected with the WT ITGA2 3′ UTR sequence, but not in cells transfected with MUT ITGA2 3′ UTR sequence. This indicates the direct binding of miR-146b-5p with the ITGA2 gene sequence (Fig. 5E). These results showed that miR-146b-5p could repress the expression of the ITGA2 gene via binding with its 3′ UTR region.

### lnc-STYK1-2 regulates bladder cancer cell processes via targeting miR-146b-5p

**Fig. 6:**
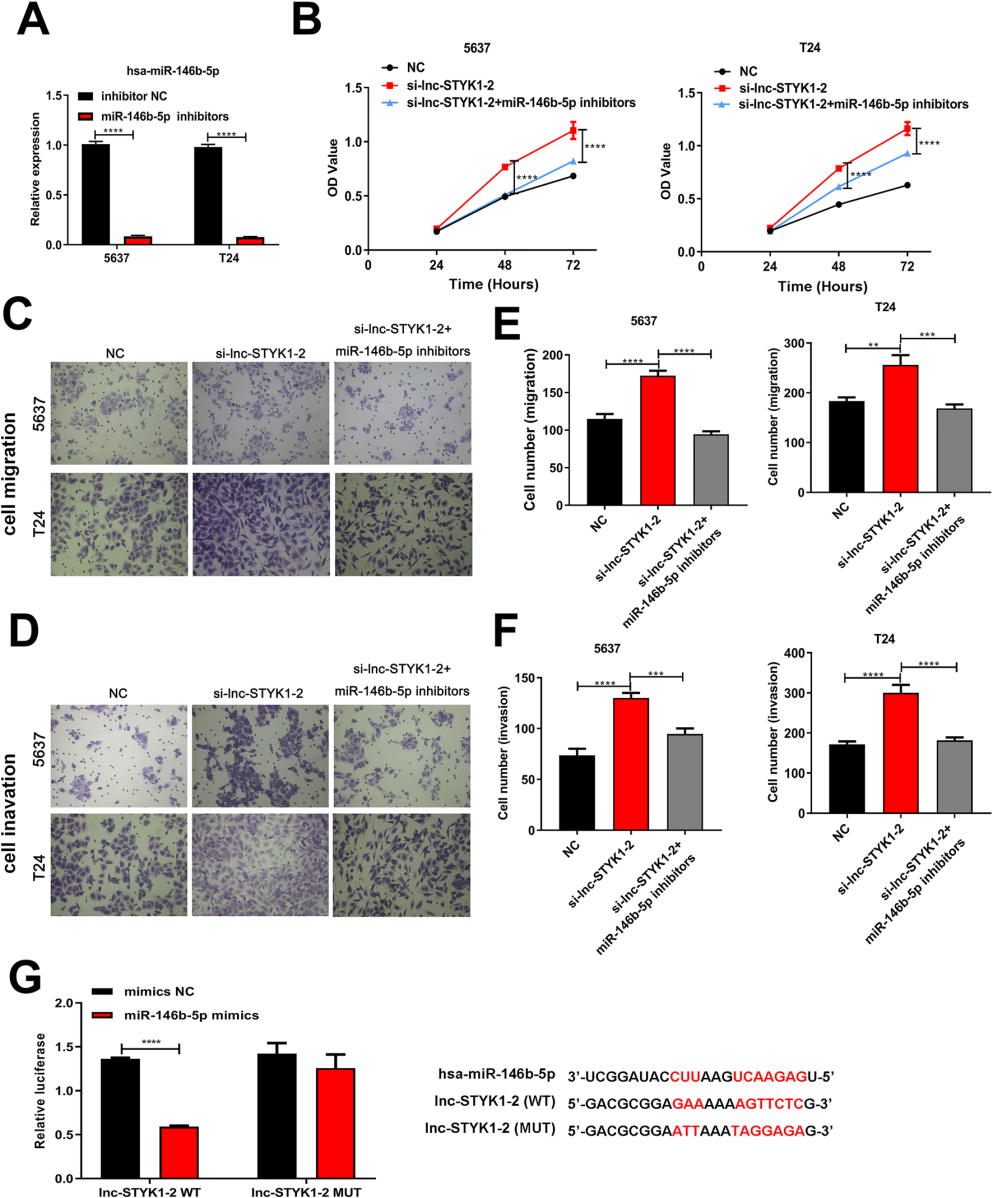
lnc-STYK1-2 regulates bladder cancer cell functions through its association with miR-146b-5P. **A** Relative expression of miR-146b-5p in 5637 and T24 cells transfected with miR-146b-5p inhibitors. The expression of miR-146b-5p was assessed by qRT-PCR. **B** The proliferation of 5637 and T24 cells after lnc-STYK1-2 silencing and miR-146b-5p inhibitor treatment. The proliferation of 5637 and T24 cells was assessed by MTS. **C**, **D** Effects of miR-146b-5p inhibitor treatment on the migration capacities of 5637 and T24 cells transfected with lnc-STYK1-2 siRNAs. **E**, **F** Influence of miR-146b-5p inhibitors on the invasion of 5637 and T24 cells with silenced lnc-STYK1-2 expression. The migration and invasion capacities of bladder cancer cells were evaluated using the Transwell system. **G** Direct association of lnc-STYK1-2 with miR-146b-5p in 293T cells. The binding between lncRNA and microRNA was tested via dual-luciferase reporter assay, and predicted binding sites were shown on the right. NC: negative control; WT: wild type; MUT: mutant type; *P < 0.05; **P < 0.01; ****P < 0.0001

We then subsequently addressed the possible mediating roles of miR-146b-5p in lnc-STYK1-2-regulated bladder cancer cell functions. At first, we showed that the miR-146b-5p inhibitors could significantly repress miR-146b-5p expression in the 5637 and T24 cells (Fig. 6A). Then, during the MTS assay, we showed that the promotion of 5637 and T24 cell proliferation by lnc-STYK1-2 silencing had been significantly abrogated by miR-146b-5p inhibitors (Fig. 6B). Moreover, treatment with miR-146b-5p inhibitors greatly repressed the migration of 5637 and T24 cells transfected with lnc-STYK1-2 siRNAs (Fig. 6C and D). Also, the increase in 5637 and T24 cell invasion rates caused by lnc-STYK1-2 siRNAs was remarkably mitigated by treatment with miR-146b-5p inhibitors (Fig. 6E and F). Finally, we verified whether lnc-STYK1-2 could directly associate with miR-146b-5p in cultured 293T cells by dual-luciferase reporter assay (Fig. 6G). Together, these results proved that the regulation of bladder cancer cell proliferation, migration, and invasion by lnc-STYK1-2 was mediated by directly binding the miR-146b-5p.

### lnc-STYK1-2 regulates ITGA2 and AKT/NFκB/STAT3 signaling in bladder cancer cell processes via targeting miR-146b-5p

**Fig. 7:**
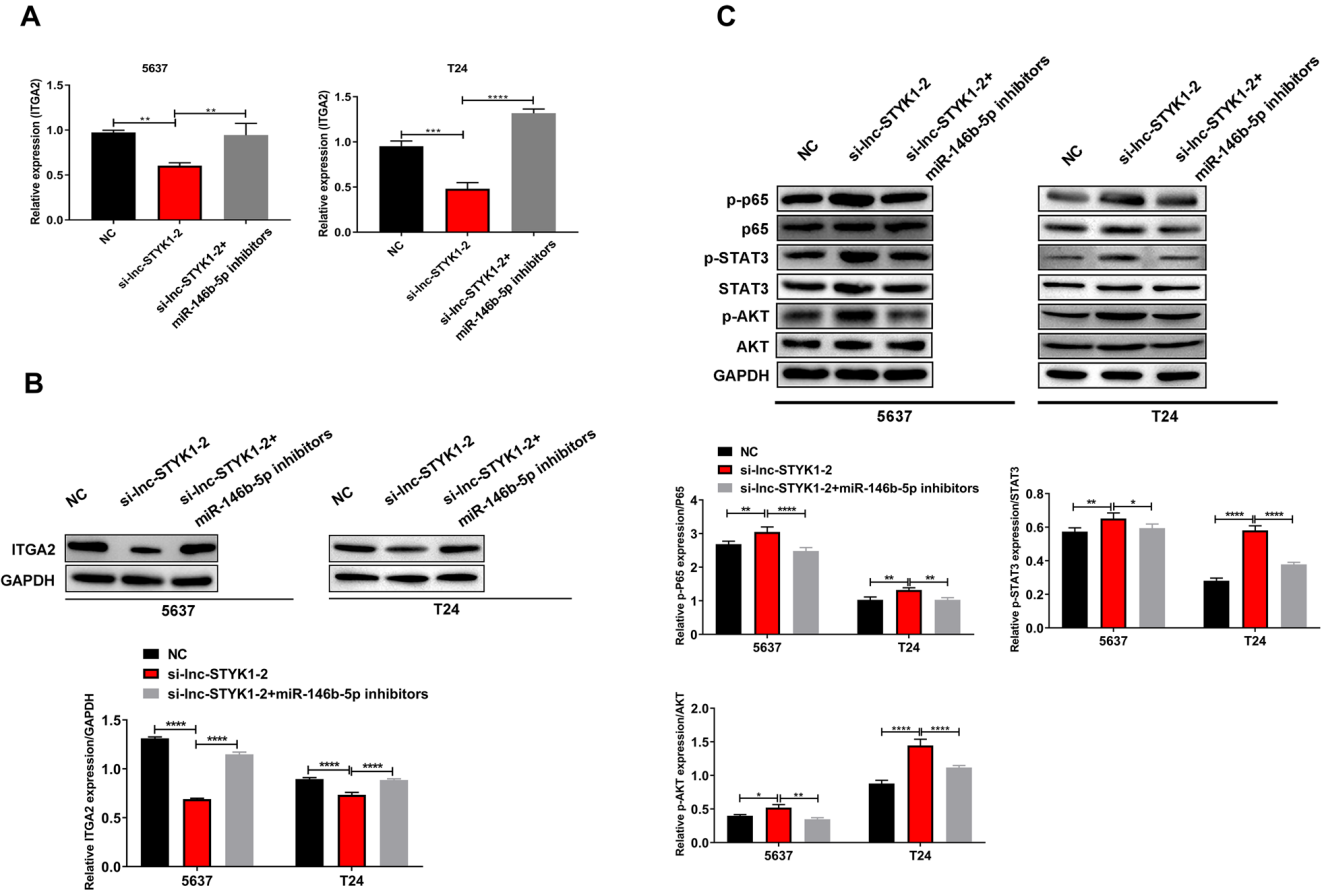
Regulation of ITGA2 and AKT/NFκB/STAT3 signaling by lnc-STYK1-2 and miR-146b-5p interaction. **A** Relative ITGA2 mRNA levels in bladder cancer cells transfected with lnc-STYK1-2 siRNAs and miR-146b-5p inhibitors. The mRNA levels were detected by the qRT-PCR assay. **B** Alterations in ITGA2 protein levels in bladder cancer cells induced by lnc-STYK1-2 siRNAs and miR-146b-5p inhibitors. ITGA2 protein contents were analyzed by Western blotting using GAPDH as the internal standard. **C** The regulative abundances of p65, STAT3, and AKT protein and their phosphorylation levels in the 5637 and T24 cells treated with lnc-STYK1-2 siRNAs and miR-146b-5p inhibitors. Western blotting was performed to analyze the expression and phosphorylation (normalized to total protein) of p65, STAT3, and AKT in bladder cancer cells. NC: negative control; ITGA2: integrin α2; GAPDH: glyceraldehyde-3-phosphate dehydrogenase; STAT3: signal transducer and activator of transcription; AKT: protein kinase B; miR-146b: miR-146b-5p; **P < 0.01; ***P < 0.001

For more understanding of the molecular mechanisms downstream of lnc-STYK1-2 and miR-146b-5p, we analyzed the influence of lnc-STYK1-2 and its suppression of miR-146b-5p on the expression of ITGA2 and AKT/NFκB/STAT3 axis activation in bladder cancer cells. By qRT-PCR, we found that the expression of the ITGA2 gene in the 5637 and T24 cells was significantly repressed by lnc-STYK1-2 siRNAs, which were then recovered by miR-146b-5p inhibitors (Fig. 7A). Also, the ITGA2 protein abundances in 5637 and T24 cells were reduced by lnc-STYK1-2 siRNAs, which were also recovered by miR-146b-5p inhibitors (Fig. 7B). Through Western blotting, we found that the phosphorylation of p65, STAT3 (signal transducer and activator of transcription), and AKT (protein kinase B) proteins in 5637 and T24 cells was greatly enhanced by lnc-STYK1-2 siRNAs compared with the control groups (Fig. 7C). However, miR-146b-5p inhibitors significantly reduced phosphorylated p65, STAT3, and AKT protein levels in 5637 and T24 cells with silenced lnc-STYK1-2 expression (Fig. 7C). Together, these results revealed that ITGA2 and AKT/NFκB/STAT3 signaling pathways were regulated by lnc-STYK1-2 and miR-146b-5p in bladder cancer cells.

## Discussion

Epigenetic modulation of functional gene expression, especially those mediated by noncoding RNAs, such as lncRNAs and microRNAs, has been established as an essential tumorigenic mechanism during the past decades [12, 24]. In bladder cancer cells, great alterations in lncRNAs and microRNAs have also been disclosed in previous reports [12, 25]; however, their pathogenic roles and underlying mechanisms remain uninvestigated. This study addressed the roles of lncRNAs in bladder cancer development by analyzing the lncRNA profile alterations through high-throughput RNA sequencing, which revealed great changes in lncRNA expression in human bladder cancer tissues. Among them, the expression of lnc-STYK1-2 was greatly downregulated in bladder cancer tissues compared with that in adjacent noncancerous tissues. Furthermore, knockdown of lnc-STYK1-2 expression significantly promoted the proliferation, migration, and invasion of bladder cancer cells and the tumorigenic capacity of cancer cells in nude mice. Regarding molecular mechanism, we further showed that lnc-STYK1-2 could directly bind with and suppress the expression of miR-146b-5p in bladder cancer cells. Finally, treatment with miR-146b-5p inhibitors greatly abrogated the regulatory effects of lnc-STYK1-2 silencing on bladder cancer cell proliferation, migration, and invasion. These investigations revealed new molecular mechanisms of bladder cancer pathogenesis involving gene expressional modulation by lncRNAs and microRNAs.

As mentioned, gene expression regulated by lncRNAs has been established as an essential pathogenic mechanism in bladder cancer development during the past years [12–14]. However, compared with many lncRNAs whose expression showed great alterations in bladder cancer cells, their functional investigations remain poorly understood. To characterize lncRNAs involved in bladder cancer progression, we first compared the lncRNA profiles between bladder cancer tissues and adjacent noncancerous bladder tissues. We showed that many lncRNAs showed a significant expressional decrease in bladder cancer tissues than the number of lncRNAs upregulated in bladder cancer tissues. This indicates the prevalent significance of lncRNA repression during bladder cancer development. STYK1 (serine/threonine/tyrosine kinase 1), also known as NOK (novel oncogenic kinase), is a member of the PTK (protein tyrosine kinase) protein subfamily closely associated with the initiation and progression of multiple human cancers [26–28]. The oncogenic role of STYK1 was also mediated by activation of downstream AKT through the catalysis of AKT protein phosphorylation at Thr308 [29]. However, little is known about the roles of STYK1 in bladder cancer development. This study showed that the lncRNA lnc-STYK1-2 encoded by the STYK1 gene serves as an essential tumor suppressor factor in bladder cancer, supported by enhanced proliferation, migration, invasion, and tumorigenesis induced by lnc-STYK1-2 silencing. These results disclosed a new layer of STYK1 functioning in bladder cancer progression, whose potential application in cancer prediction, diagnosis, and treatment deserves further investigation.

The major biological and pathogenic roles of lncRNAs have been reported to be mediated by their interference with gene expression through association with transcription-repressing microRNAs [30]. For insights into the microRNA-mediated molecular mechanism downstream of lnc-STYK1-2, we established a lncRNA–microRNA–mRNA interaction network based on lnc-STYK1-2 and differentially expressed mRNA and found that miR-146b-5p might act as a ceRNA for lnc-STYK1-2. Previous reports showed that miR-146b-5p was involved in developing multiple human cancers, such as nasopharyngeal carcinoma and bladder cancer, targeting functional genes, such as MMP2 and ETS2 [20, 21]. This study confirmed the direct association of lnc-STYK1-2 with miR-146b-5p by dual-luciferase activity, further supported by the elevated miR-146b-5p expression in bladder cancer cells with lnc-STYK1-2 silencing. Notably, we showed that miR-146b-5p inhibitors could substantially abrogate the effects of lnc-STYK1-2 silencing on bladder cancer cell proliferation, migration, and invasion capacities, which proved the pathogenic roles of lnc-STYK1-2/miR-146b-5p interaction during bladder cancer development and progression. Additionally, the tumorigenic effects of miR-146b-5p were previously reported to be regulated by other lncRNAs, such as lncRNA NEAT1, SOX2-OT, and lnc-AL445665.1–4 [20, 31, 32]. Therefore, the possibility of miR-146b-5p targeting by other cancer-related lncRNAs in bladder cancer cells remains to be explored.

Cancer pathogenesis is also closely controlled by the collaborative regulation of multiple classical signaling cascades. For instance, the integrin subunit ITGA2 was previously shown to promote cancer cell proliferation, invasion, and drug resistance by activating AKT signaling pathways [33]. Moreover, the phosphorylation and activation of p65 protein, a major component of the NF-κB complex, essentially contributed to enhanced bladder cancer cell proliferation, growth, and responses to therapeutic reagents [34]. However, activating the NF-κB signaling pathway during cancer development and other pathogenic processes also involves phosphorylating AKT and STAT3 proteins [34–36]. Moreover, the activation of AKT and NF-κB signaling pathways could be regulated by the miR-146b-5p, which is linked with glioma cell proliferation and apoptosis, and survival of patients with glioma [37], but little is known about the epigenetic regulation of ITGA2 and the ATK/NF-kB/STAT3 axis in bladder cancer cells. This study proved that the ITGA2 expression could be directly targeted and suppressed by miR-146b-5p. Also, ITGA2 expression and phosphorylation of AKT, STAT3, and p65 proteins in bladder cancer cells were greatly enhanced by silencing of lnc-STYK1-2 expression. Notably, the elevation of ITGA2 expression, AKT, STAT3, and p65 protein phosphorylation by lnc-STYK1-2 silencing was effectively mitigated by miR-146b-5p inhibitor treatment. These results have established the ITGA2 and ATK/NF-kB/STAT3 cascade as a key signaling pathway downstream of lnc-STYK1-2/miR-146b-5p interaction during the initiation and progression of bladder cancer.

Considering the essential functions of lnc-STYK1-2 and miR-146b-5p interaction revealed here in bladder cancer cells, their potential for future applications in diagnosing or treating patients with cancer requires further evaluation through large-scale pre-clinical experiments. Furthermore, many lncRNAs were shown to be differentially expressed in the bladder cancer tissues, as shown in this study. Notably the roles of other differentially expressed lncRNAs in regulating bladder cancer cell activities and their associations with bladder cancer progression and prognosis deserve further investigation. Finally, the pathogenic roles of ITGA2 in bladder cancer cells should also be further explored using cellular and animal models.

## Conclusion

In summary, by combining RNA sequencing and functional investigation, we discovered that lnc-STYK1-2 acts as a new tumor suppressor of bladder cancer that repressed bladder cancer cell proliferation, migration, invasion, and tumorigenesis by targeting miR-146b-5p to modulate ITGA2 and the ATK/STAT3/NF-kB signaling pathway. These findings put a new perspective on the epigenetic landscapes driving bladder cancer initiation and development, which could be further explored as new targets for bladder cancer prevention, diagnosis, and targeted treatment.

## Data Availability

The data that support the findings of this study are available from the corresponding author upon reasonable request.
